# Child Support Grant access and receipt among 12-week-old infants in an urban township setting in South Africa

**DOI:** 10.3402/gha.v7.25310

**Published:** 2014-08-25

**Authors:** Wanga Zembe-Mkabile, Tanya Doherty, David Sanders, Debra Jackson

**Affiliations:** 1 Health Systems Research Unit, South African Medical Research Council, Cape Town, South Africa; 2 School of Public Health, University of the Western Cape, Cape Town, South Africa; 3 Knowledge Management & Implementation Research Unit Health Section, UNICEF, New York, USA

**Keywords:** cash transfers, child health, take-up rates, Child Support Grant, South Africa

## Abstract

**Background:**

Cash transfers (CTs) are increasingly used as a strategy to alleviate poverty and improve child health outcomes in low- and middle-income countries. The Child Support Grant (CSG) is the largest CT programme in South Africa, and on the continent, targeting poor children from birth until the age of 18 with a monthly sum of R300 (USD30). Evidence on the CSG shows that early receipt of the grant is associated with improved child health outcomes. Since its implementation, one of the major concerns about the grant has been take-up rates, particularly for younger children. This paper reports results on take-up rates for 12-week-old infants residing in an urban township in South Africa.

**Methods:**

This is a descriptive study utilising data from a community-based, cluster-randomised trial which evaluated a programme providing pregnancy and post-natal home visits by community health workers to 3,494 mothers in Umlazi township, South Africa.

**Results:**

At the 12-week visit, half (52%) of the mothers who had enrolled in the study had applied for the CSG on behalf of their children, while 85% of the mothers who had not applied were still planning to apply. Only 38% (1,327) of all children had received the CSG.

**Conclusions:**

In this study, many mothers had not applied for the CSG in the first few months after delivery, and only a third of children had accessed the grant. Further research is needed to understand what the current barriers are that prevent mothers from applying for this important form of social protection in the early months after delivery.

In recent years, cash transfer (CT) programmes have become popular policy instruments for reducing child poverty and improving a range of child health outcomes in low- and middle-income countries (1–3). Such CT programmes can take the form of conditional or unconditional cash grants, can be means-tested, and targeted or universal (4, 5). Over the past decade, a strong evidence base has been built in low- and middle-income countries to show that CTs, even if transferring small amounts of money to poor children, can have a positive impact on child growth and nutritional status (6–8), school attendance and educational attainment (9, 10), reduce child hunger, and reduce risky sexual behaviours amongst adolescent children (11–13). This strong evidence base has increased the urgency and call for countries to expand child-focused CT programmes, and, where take-up is low, to accelerate it.

In South Africa, the Child Support Grant (CSG) is the largest CT programme in the country and on the continent. It targets children from poor households who receive about USD30 per month from birth until the age of 18. To qualify for the grant, caregivers of CSG applicants have to earn less than 10 times the amount of the CSG (currently this amounts to <R3600 per year or <USD360 per year for single caregivers, and <R7200 per year or <USD720 per year for married or cohabiting couples) (14).

Since its implementation, one of the major concerns about the CSG has been take-up rates, particularly for younger children (15–17). More recent evidence has reported take-up rates of about 60% for children under 2 years (9, 17), and even lower take-up rates for children under 6 months (17). Children who receive the CSG early after birth have been shown to achieve better growth (height-for-age) and nutritional status than children who receive it later (7).

## Methods

This paper reports results on CSG access, reasons for non-application and non-receipt among caregivers of 12-week-old children in Umlazi, a large urban township in Durban, South Africa. The study utilised data from a community-based, cluster-randomised trial which evaluated a programme providing pregnancy and post-natal home visits by community health workers (CHWs) to encourage exclusive breastfeeding (18). In the control arm, mothers received home visits (one antenatal and two post-natal) from CHWs who advised them on the process of applying for a CSG; however, the CHW visits in the control arm had no effect on grant uptake at 12 weeks (relative risk 0.97; 95% CI: 0.90–1.03). Details of the trial are published elsewhere (18, 19).

The main outcomes of the trial were measured at 12 weeks. Questions related to the CSG were included in the questionnaire with self-reported responses regarding whether a mother had applied for the CSG, whether they were intending to apply, and reasons for not applying. To apply for the CSG, a mother or primary caregiver of the child for whom the grant is sought needs to have the following documents: an identity document (ID) for the mother or primary caregiver, birth certificate of the child, and proof of income (or an affidavit confirming unemployment).

### Ethical considerations

The analysis of CSG take-up was included in the trial protocol submitted for ethical approval. The Medical Research Council ethics review board (EC08-002) approved the trial study. A Community Advisory Board (CAB), consisting of local stakeholders, was established and acted as a liaison between the community and research staff.

## Results

### Application for receipt of the CSG

**Fig. 1:**
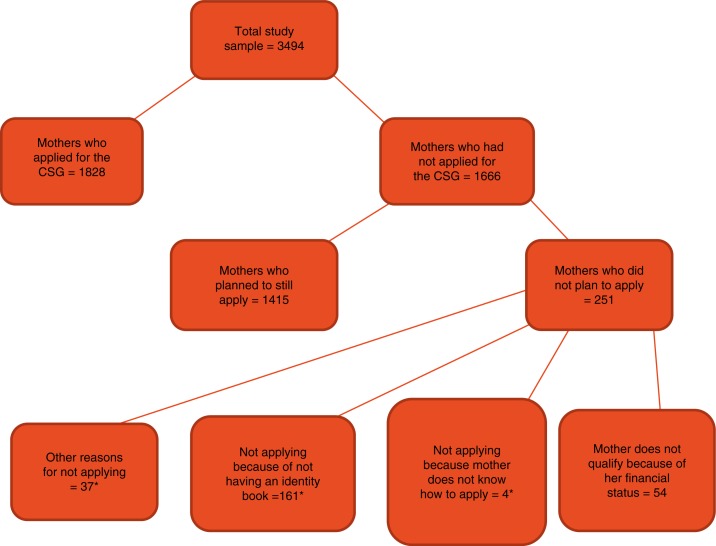
CSG application and reasons for not applying. *Five mothers gave more than one reason for not applying.

Figure 1 presents data on indicators of CSG application and receipt. Among the 3,494 children whose caregivers were interviewed at 12 weeks, 1,828 (52%) had applied for the CSG. A large proportion of mothers did have the required documents needed for the application with 86% of mothers possessing an ID book and 73% of infants having a birth certificate. Amongst mothers who had not applied, 1,415 (85%) reported that they still intended to apply for the grant. Reasons for not applying for the CSG ranged from not possessing an ID document (161/251, 64%), to not qualifying for the CSG because of reported financial status that mothers perceived to be higher than the official means-tested threshold (54/251, 21%). Given the importance of early receipt, it is concerning that almost half (48%) of the mothers in this study had still not applied for the grant 3 months after giving birth.

Very few mothers attributed not applying for the CSG to lack of knowledge about the application process (4/251, 1.5%). These results are encouraging as they are in sharp contrast to the situation in the early years after the CSG was implemented, where many eligible caregivers did not apply for the CSG because they did not know what the process entailed (15, 20), and where not possessing a birth certificate was one of the major administrative barriers to CSG receipt (17).

### CSG receipt

**Fig. 2:**
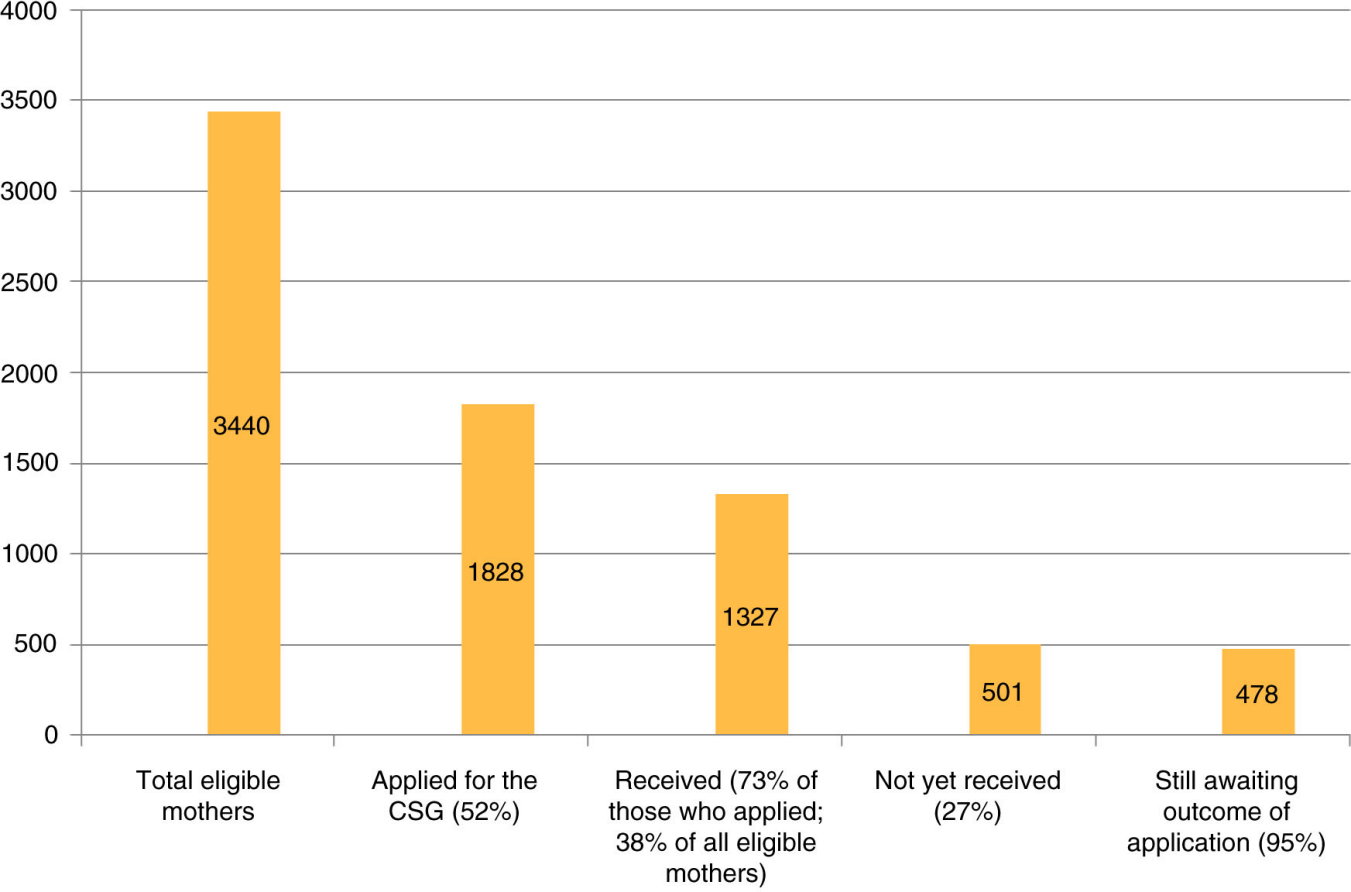
Cascade showing CSG receipt at 12 weeks post-delivery.

Of the 1,828 children who applied for the CSG, 1,327 (73%) had received it at 12 weeks. However, in the total sample only 38% were in receipt of the grant (Fig. 2). Among the 501 non-recipients, 478 (95%) were not in receipt of the CSG at 12 weeks because they were still waiting for their applications to be processed. The turn-around time for CSG receipt is 21 working days after an application has been submitted, and thus a number of these applicants could have still been within that window period.

## Conclusion

While knowledge about the CSG application process seems to have improved since the early years of the grant's implementation, this study has shown that overall only 38% of children, the great majority of whom are likely to have been eligible for the CSG, were in receipt of the grant by 12 weeks of age, despite the majority of mothers possessing the administrative requirements (ID book and birth certificate). From these results, it is clear that the major reason for low receipt in this group of infants is that mothers initiate application for the CSG late. Further qualitative research is needed to understand what the current barriers are that prevent mothers from applying for this important form of social protection in the early months after delivery.
